# Dysregulation of the Tryptophan Pathway Evidences Gender Differences in COPD

**DOI:** 10.3390/metabo9100212

**Published:** 2019-10-01

**Authors:** Shama Naz, Maria Bhat, Sara Ståhl, Helena Forsslund, C. Magnus Sköld, Åsa M. Wheelock, Craig E. Wheelock

**Affiliations:** 1 Division of Physiological Chemistry 2, Department of Medical Biochemistry and Biophysics, Karolinska Institutet, SE 171 77 Stockholm, Sweden; shamanishu@yahoo.com; 2 Research and Development, Innovative Medicines, Personalised Healthcare and Biomarkers, Translational Science Centre, Science for Life Laboratory, AstraZeneca, SE 171 65 Solna, Sweden; Maria.Bhat@astrazeneca.com (M.B.); sara.stahl@me.com (S.S.); 3 Department of Clinical Neuroscience, Karolinska Institutet, SE 171 77 Stockholm, Sweden; 4 Respiratory Medicine Unit, Department of Medicine Solna & Center for Molecular Medicine, Karolinska Institutet, SE 171 77 Stockholm, Sweden; helena.forsslund@gmail.com (H.F.); Magnus.Skold@ki.se (C.M.S.)

**Keywords:** tryptophan, kynurenine, serotonin, gender, smoking, IDO, COPD

## Abstract

Increased activity of indoleamine 2,3-dioxygenase (IDO) and tryptophan hydroxylase (TPH) have been reported in individuals with chronic obstructive pulmonary disease (COPD). We therefore investigated the effect of gender stratification upon the observed levels of tryptophan metabolites in COPD. Tryptophan, serotonin, kynurenine, and kynurenic acid were quantified in serum of never-smokers (*n* = 39), smokers (*n* = 40), COPD smokers (*n* = 27), and COPD ex-smokers (*n* = 11) by liquid chromatography coupled with tandem mass spectrometry (LC–MS/MS). The individual metabolite associations with lung function, blood, and bronchoalveolar lavage (BAL) immune-cell composition, as well as chemokine and cytokine levels, were investigated. Stratification by gender and smoking status revealed that the observed alterations in kynurenine and kynurenic acid, and to a lesser extent serotonin, were prominent in males, irrespective of COPD status (kynurenine *p* = 0.005, kynurenic acid *p* = 0.009, and serotonin *p* = 0.02). Inferred serum IDO activity and kynurenine levels decreased in smokers relative to never-smokers (*p* = 0.005 and *p* = 0.004, respectively). In contrast, inferred tryptophan hydroxylase (TPH) activity and serotonin levels showed an increase with smoking that reached significance with COPD (*p* = 0.01 and *p* = 0.01, respectively). Serum IDO activity correlated with blood CXC chemokine ligand 9 (CXCL9, *p* = 0.0009, *r* = 0.93) and chemokine (C-C motif) ligand 4 (CCL4.(*p* = 0.04, *r* = 0.73) in female COPD smokers. Conversely, serum serotonin levels correlated with BAL CD4+ T-cells (%) (*p* = 0.001, *r* = 0.92) and CD8+ T-cells (%) (*p* = 0.002, *r* = −0.90) in female COPD smokers, but not in male COPD smokers (*p* = 0.1, *r* = 0.46 and *p* = 0.1, *r* = −0.50, respectively). IDO- and TPH-mediated tryptophan metabolites showed gender-based associations in COPD, which were primarily driven by smoking status.

## 1. Introduction

Chronic obstructive pulmonary disease (COPD) is characterized by persistent, largely irreversible airway obstruction caused by chronic inflammation [1,2]. The inflammatory response to cigarette smoke is the major etiological factor, and is an important component driving both the onset of COPD and associated tissue damage in the majority of patients [3]. Previous studies have shown a profound impact of cigarette smoke on T-cells and their release of pro-inflammatory mediators in COPD [4,5,6,7,8].

**Figure 1:**
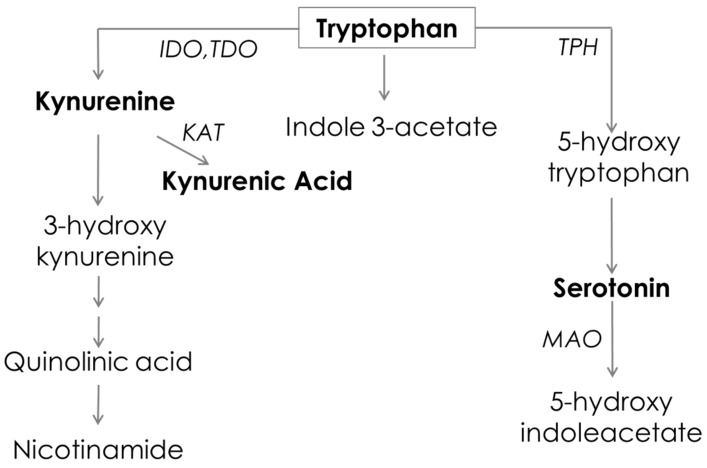
Tryptophan pathway. Metabolites marked in bold were quantified by LC-MS/MS in the current study. IDO = indoleamine 2,3-dioxygenase, MAO = monoamine oxidase, TDO = tryptophan 2,3-dioxygenase, KAT = kynurenine aminotransferase, TPH = tryptophan hydroxylase.

Tryptophan is an essential amino acid that is metabolized via multiple biosynthetic pathways in numerous cell types [9]. In addition, the microbial metabolism of tryptophan can produce a suite of downstream metabolites, highlighting the role of the microbiome in tryptophan metabolism [10]. Tryptophan and its metabolites have been studied in inflammatory and respiratory disease, and found to be altered during infections as part of the immune reaction [11,12,13,14]. Tryptophan breakdown occurs via two major pathways; ~95% of tryptophan is metabolized via the kynurenine pathway and the other ~5% is metabolized via the serotonin pathway (Figure 1) [15,16]. Indoleamine 2, 3-dioxygenase (IDO) is the rate-limiting enzyme in the kynurenine pathway. The serotonin pathway is catalyzed by tryptophan hydroxylase (TPH), and is further metabolized by monoamine oxidase (MAO) [17]. IDO is also able to suppress oxidative stress by using superoxide anions as both the substrate and cofactor in its catalytic process [18], while inhibiting T-cell proliferation [19]. IDO has been studied in COPD, and therapeutics targeting IDO have been shown to improve COPD symptoms [20]. Recent findings suggested the involvement of serotonin in the pathogenesis of COPD; serotonin plays an important role in pulmonary vasoconstriction and bronchoconstriction [11,21]. Furthermore, cigarette smoke has been shown to inhibit MAO, leading to elevated serotonin levels [22].

The Karolinska ‘COPD & Smoking from an “OMIC” Perspective’ (COSMIC) cohort was designed to investigate the role of gender in mild-to-moderate COPD [4,23,24,25]. A previously published metabolomics study identified a set of tryptophan pathway metabolites to be dysregulated in COPD ([22], Figure S1). Accordingly, the aim of the current study was to further investigate the finding using a quantitative liquid chromatography tandem mass spectrometry (LC-MS/MS) method and to investigate the association of IDO and TPH pathways in the immunomodulatory effects of smoking and COPD. While previous studies have examined tryptophan metabolites and their relation to COPD [11,13,26,27,28], none performed gender stratification.

## 2. Results

### 2.1. Serum Levels of the Tryptophan Pathway Metabolites

**Figure 2:**
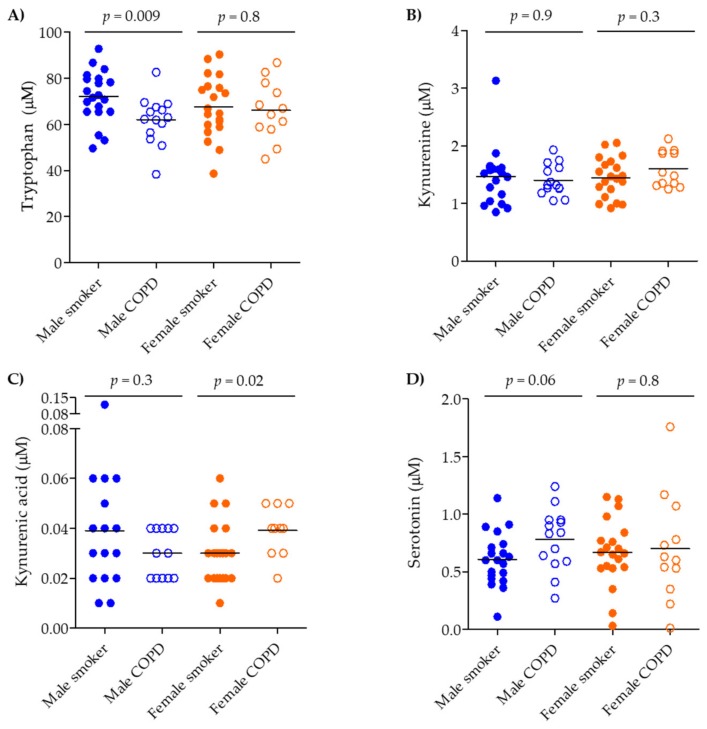
Serum concentration of tryptophan pathway metabolites in smokers vs. COPD smokers, stratified by gender. (**A**) tryptophan, (**B**) kynurenine, (**C**) kynurenic acid, and (**D**) serotonin. Groups: Male smokers (*n* = 20), Male COPD (*n* = 14), Female smokers (*n* = 20), Female COPD (*n* = 12). Smokers: closed circles, COPD smokers: open circles, Male: blue, Female: orange. Significance was tested by applying a non-parametric Mann–Whitney test.

The median (interquartile range, IQR) serum levels of tryptophan, serotonin, kynurenine, and kynurenic acid are shown in Table 1. The quantified levels of tryptophan and metabolites were in the expected range for human serum [29]. The acquired targeted data correlate well with the previously published metabolomics data ([22], Figure S2). All compounds were detected in all subjects, except for kynurenic acid in one smoker and kynurenine in one COPD ex-smoker, which were below the limit of quantitation (LOQ) and therefore the missing values were replaced with one-third of the LOQ for statistical analysis. Tryptophan itself did not show any significant alterations across all four groups (ANOVA *p* = 0.2; Figure S3A, Table 1); however, gender stratification identified a decrease in male COPD smokers compared to male smokers (*p* = 0.009) that was not observed in the corresponding females, (*p* = 0.8; Figure 2A).

**Figure 3:**
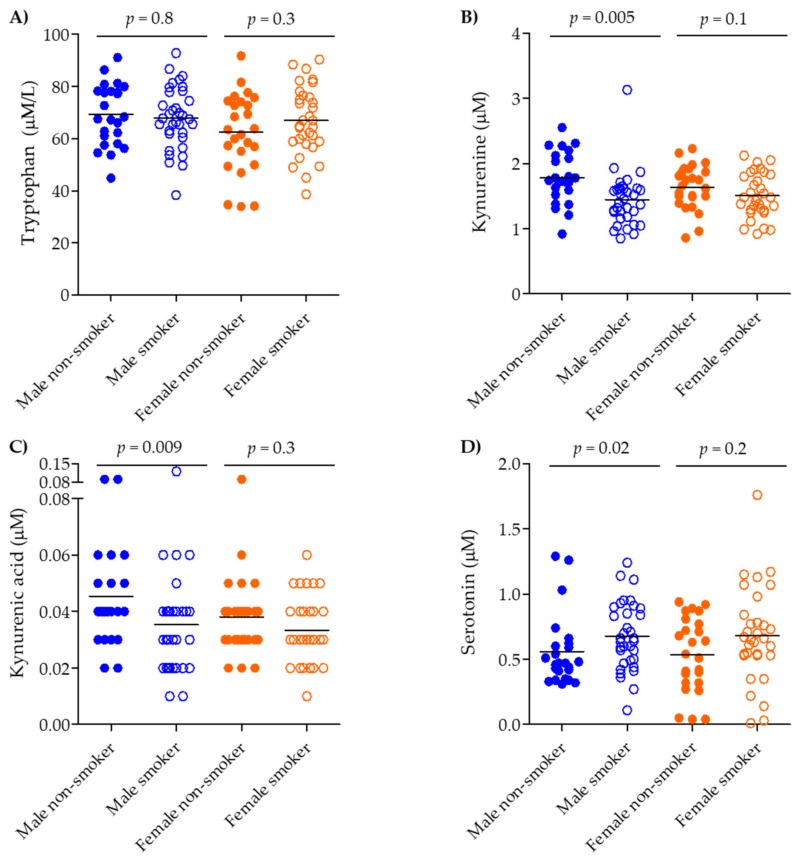
Serum concentration of tryptophan pathway metabolites in combined non-smokers vs. smokers irrespective of COPD status, stratified by gender. (**A**) tryptophan, (**B**) kynurenine, (**C**) kynurenic acid, and (**D**) serotonin. Groups: Male non-smokers (*n* = 24), Male smokers (*n* = 34), Female non-smokers (*n* = 25), Female smokers (*n* = 32). Smokers: closed circles, COPD smokers: open circles, male: blue, female: orange. Significance was tested by applying a non-parametric Mann–Whitney test. The corresponding *p*-values for the non-smoker vs. smoker combined gender comparisons were *p* = 0.6, *p* = 0.004, *p* = 0.008, and *p* = 0.009, respectively.

The IDO pathway evidenced smoking- and COPD-related shifts. Kynurenine was shifted across all groups (Figure S3B; ANOVA *p* = 0.003), decreasing in smokers (*p* = 0.004) and COPD smokers (*p* = 0.02) compared to never-smokers (Figure S3B); however, it remained unchanged between smokers and COPD smokers (*p* = 0.2). Stratifying by gender and current smoking status irrespective of COPD diagnosis revealed that the differences were driven mainly by male gender and smoking (males: non-smokers vs. smokers, *p* = 0.005; females: non-smokers vs. smokers, *p* = 0.1, Figure 3B). Kynurenic acid was also dysregulated across all groups (ANOVA *p* = 0.009), but was driven solely by an increase in the COPD ex-smokers group (vs. smokers, *p* = 0.02 and vs. COPD smokers, *p* = 0.002) (Figure S3C). Gender stratification showed that kynurenic acid was increased in female COPD smokers vs. smokers (*p* = 0.02, Figure 2C). Following stratification based on smoking and gender (irrespective of COPD status), kynurenic acid showed significant decrease in the male population when comparing male non-smokers to smokers (*p* = 0.009, Figure 3C).

The TPH pathway showed the opposite alteration relative to the IDO pathway. Serotonin was increased across all groups (ANOVA *p* = 0.03), increasing in COPD smokers vs. never-smokers (*p* = 0.01; Figure S3D). Gender and smoking stratification (irrespective of COPD status) again revealed that the contribution was driven by the smoking male population (male non-smokers vs. smokers, *p* = 0.02; female non-smokers vs. smokers *p* = 0.2; Figure 3D).

**Figure 4:**
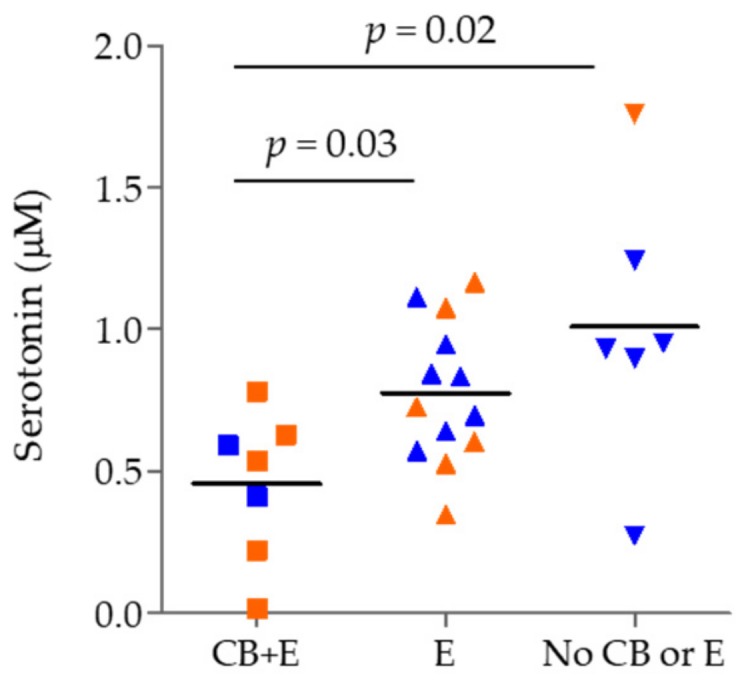
The level of serotonin in the current smoker COPD group stratified by chronic bronchitis (CB), emphysema (E) and absence of CB and E. Male: blue, Female: orange. Significance was tested using a non-parametric Mann–Whitney test.

The COPD smoking group was also stratified by chronic bronchitis (CB), emphysema (E), and/or neither (no CB nor E). Among all four compounds, only serotonin evidenced significant alterations among the three groups (*p* = 0.02; Figure 4). Due to the small number of subjects, stratification by gender could not be performed for this comparison, but gender is indicated by color-coding in the figure.

### 2.2. Serum IDO and TPH Activity

**Figure 5:**
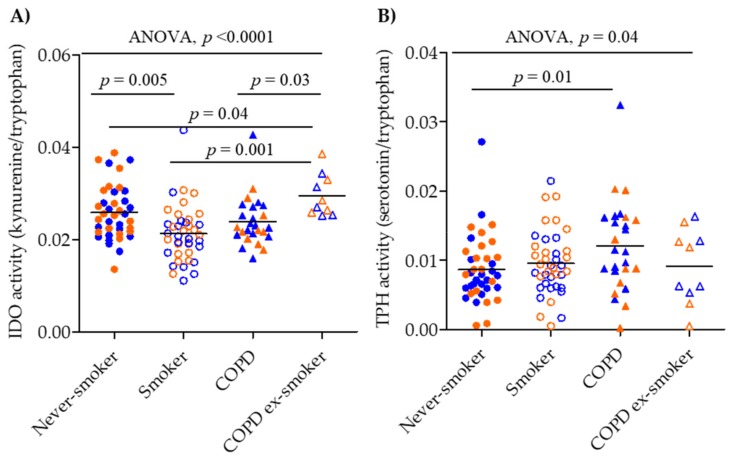
(**A**) IDO activity was estimated by the ratio of kynurenine/tryptophan concentrations and (**B**) TPH activity was estimated by the ratio of serotonin/tryptophan concentrations. Never-smokers: closed circles, smokers: open circles, COPD smokers: closed triangles, COPD ex-smokers: open triangles. Male: blue, Female: orange. Significance was tested by applying a non-parametric Kruskal–Wallis one-way ANOVA and a non-parametric Mann–Whitney test.

Inferred IDO activity was reduced due to both smoking and COPD disease status (Figure 5). The most significant alteration was observed in smokers compared to never-smokers (*p* = 0.005) (Figure 5A), but also the comparison of current-smoker and ex-smoker COPD patients was significant (*p* = 0.03). Although it did not reach significance, the median serum IDO activity was elevated in COPD smokers compared to smokers (*p* = 0.06). Gender stratification did not affect the observed differences; similar trends were seen in both males and females. Serum IDO activity was also plotted according to the COPD Global Initiative for Obstructive Lung Disease (GOLD) stage, and found to decrease with increasing disease severity (*p* = 0.03, Figure S4).

Serum TPH activity was inferred by the ratio of serotonin/tryptophan and was elevated in COPD smokers compared to never-smokers (*p* = 0.01; Figure 5B). Gender stratification showed that TPH increased in the male smoking and COPD smoking population compared to male never-smokers (never-smokers vs. COPD smokers, *p* = 0.01; smokers vs. COPD smokers, *p* = 0.02; Figure S5).

### 2.3. Correlation of Serum Tryptophan Metabolites and IDO with Clinical Data

No significant correlations were observed between age, body mass index (BMI), lung function and pack-years of smoking with serum levels of any of the four metabolites or the indices of IDO and TPH activity (data not shown). Serum IDO correlated with specific cytokine- and chemokine levels in blood and BAL fluid (Table 2). Using a threshold of significance of *p* <0.05 and |*r*| >0.5, serum IDO positively correlated with blood CXC chemokine ligand 9 (CXCL9, *p* = 0.009, *r* = 0.59) and chemokine (C-C motif) ligand 4 (CCL4, *p* = 0.02, *r* = 0.56), as well as with CD4+ forkhead box P3 (FOXP3)+ broncheoalveolar lavage (BAL) cells (*p* = 0.01, *r* = 0.57) in COPD smokers (Table 2). Following stratification by gender, the positive correlations remained significant only in female COPD smokers for CXCL9 (*p* = 0.0009, *r* = 0.93) and CCL4 (*p* = 0.04, *r* = 0.73), and were not significant in the corresponding male population. Some additional chemokines and cytokines showed a significant positive association with female COPD smokers following stratification by gender, including CXCL10 (*p* = 0.004, *r* = 0.88), CXCL12 (*p* = 0.02, *r* = 0.73), CCL3 (*p* = 0.002, *r* = 0.88), interleukin (IL)-12 (*p* = 0.05, *r* = 0.67), and IL-13 (*p* = 0.01, *r* = 0.81) (Table 2).

**Figure 6:**
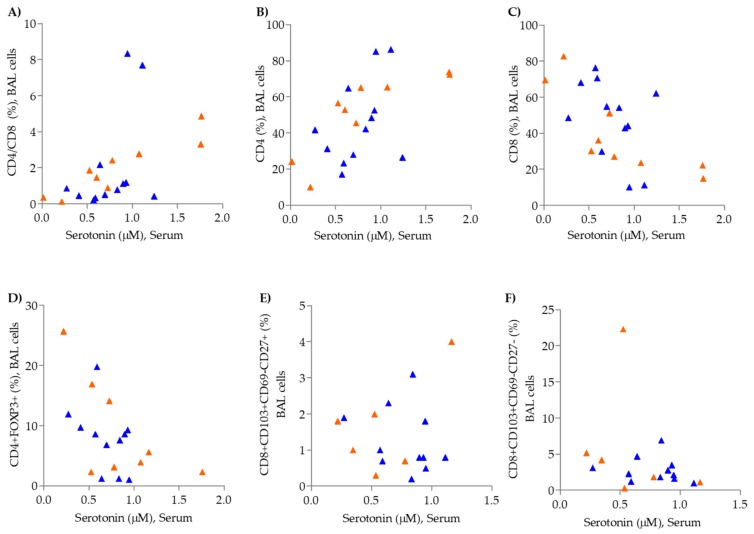
Correlation of serum serotonin levels with T-cell populations from BAL cells in COPD smokers. (**A**) CD4/CD8 %, (**B**) CD4 %, (**C**) CD8 % (**D**) CD4+ FOXP3+ %, (**E**) CD8+ CD103+ CD69− CD27+ % and (**F**) CD8+ CD103+ CD69− CD27− %. Male: blue triangles, Female: orange triangles.

Serum serotonin levels correlated positively with BAL CD4+ T-cells (%) (*p* = 0.001, *r* = 0.65) and negatively correlated with BAL CD8+ T-cells (%) (*p* = 0.001, *r* = −0.65) in COPD smokers (Figure 6A–C); however, levels did not correlate with blood CD4+ T-cells (%) or CD8+ T-cells (%) (Figure S6). Gender stratification revealed that the correlation was female-driven (female: BAL CD4+ T-cells (%), *p* = 0.001, *r* = 0.92, and BAL CD8+ T-cells (%), *p* = 0.002, *r* = –0.90; male: BAL CD4+ T-cells (%), *p* = 0.1, *r* = 0.46, and BAL CD8+ T-cells (%), *p* = 0.1, *r* = –0.50).

Significant correlations were also found in BAL CD4+ and CD8+ T-cell subsets in the COPD smoker group with serotonin: CD4+ FOXP3+ (*p* = 0.02, *r* = -0.53), CD8+ CD103+ CD69− CD27+ (*p* = 0.03, *r* = 0.53), and CD8+ CD103+ CD69− CD27− (*p* = 0.04, *r* = 0.50) (Figure 6D–F). Serotonin levels also showed a positive association with emotional function in female COPD smokers (*p* = 0.03, *r* = 0.64), but not males (*p* = 0.1, *r* = −0.45, (Figure S7). The emotional function was ascertained from the CRQ (Chronic Respiratory Disease Standardized Questionnaire), which addresses quality of life based upon six domains including “emotional function” as previously reported by Forsslund et al. [4].

## 3. Discussion

A previous exploratory study that applied metabolomics to the Karolinska COSMIC cohort reported an association between tryptophan pathway metabolites, smoking and COPD [23]. Out of the nine identified tryptophan pathway metabolites, the abundance of kynurenine decreased and serotonin increased in healthy smokers and/or COPD smokers compared to the never-smoking population (Figure S1). In order to examine these associations in greater detail, the current study quantified the levels of tryptophan metabolites using a targeted assay. The targeted quantification confirmed the findings from the previous metabolomics study, further highlighting the potential role of the tryptophan pathway in smoking and COPD.

Although tryptophan levels did not evidence any significant alterations in joint gender comparisons, gender stratification revealed that tryptophan levels decreased in male smokers and COPD smokers relative to never-smokers (Figure S8). However, no major gender differences in tryptophan metabolism were observed in the never-smoking population, with only kynurenic acid evidencing a small increase in males (*p* = 0.04). A few studies have previously reported reduced blood tryptophan levels and increased kynurenine, suggesting an increase in IDO activity in pneumonia patients [30,31]. Elevated IDO activity was also reported in acute exacerbations of COPD at the time of hospitalization, and then shown to return to lower levels at the one month follow-up [13,32]. In the current study, serum IDO activity was decreased in smokers relative to never-smokers, but increased with COPD diagnosis. These findings are in agreement with previous reports from a population-based study of smokers which also evidenced decreased serum IDO activity compared to a never-smoking population postulating that components of cigarette smoke may potentially exert immunosuppressive effects upon serum IDO activity [33]. Interestingly, our study showed diminished estimated serum IDO activity not only in smokers, but also in COPD smokers compared to never-smokers, which is in line with previous findings by Pertovaara et al. [33].

In contrast, a progressive reduction of sputum IDO activity in smoking and smoking COPD patients has been reported, postulating a chronic airway neutrophilic inflammation in COPD [27]. In a follow-up study with simvastatin treatment, an enhanced expression of the airway immunomodulating enzyme IDO was observed [20]. It should be stressed that the study by Maneechotesuwan et al. included almost entirely males, comparing subjects with mixed current-smoker status both for controls (100% male; 84% ex-smokers) and individuals with COPD (86.4% male; 84% ex-smokers) [27]. Conversely, our study was gender balanced with smokers (50% male), COPD smokers (51% male), and COPD ex-smokers (50% male), which were analyzed separately to focus on smoking-related effects. The Maneechotesuwan et al. study is in agreement with our findings, which only showed significant decreases in serum tryptophan in male COPD patients compared to the healthy smoking population: However, IDO activity increased for both the genders (the median serum IDO activity was elevated in COPD smokers compared to smokers, *p* = 0.056). This divergent response between serum and sputum IDO activity in COPD smokers may potentially be due to the male-bias in the cohort.

Cigarette smoke-induced inflammation in the airways and lung parenchyma plays an important role in the pathogenesis of COPD [34], with 80% of COPD patients having a history of smoking. During inflammation, epithelial cells and macrophages are stimulated by interferon-γ (IFN-γ) release of the chemokines CXC-chemokine ligand 9 (CXCL9), CXCL10, and CXCL11, along with macrophage inflammatory protein CCL3, and CCL4. This cascade eventually stimulates CXC-chemokine receptor 3 (CXCR3), resulting in a persistent inflammatory activation in COPD [35,36]. The inflammatory cytokine INF-γ is a potent activator of IDO during inflammation via its anti-inflammatory action [37]. The positive association of IDO with CXCL9 and with the COPD smokers with CXCL9 (Table 2) suggests a potential anti-inflammatory action in response to inflammation in the COPD smokers group. However, when stratified by gender, CXCL9 along with other ligands (CXCL10, CXCL11, CCL3, and CCL4) also showed a positive correlation with IDO, but only in female COPD smokers. One possible explanation as to why women are more susceptible to the effects of tobacco smoke is a potential dose-dependent effect [38]. The airways of women are smaller and thus each cigarette represents a proportionately greater exposure. Female COPD smokers are therefore more vulnerable to inflammation and release of cytokines and chemokines [38]. Moreover, chemokines are potent chemo-attractants and reactive oxygen species have been observed in vitro after chemokine stimulation [39,40]. It has been well established that dysregulation in anti-oxidative stress pathways is associated with COPD in females [41]. In particular, we have previously reported an increase in oxidative stress-associated pathways in the female population of the COSMIC study [23]. The positive interaction of chemokines with IDO activity in only the female COPD population suggests elevated oxidative stress, strengthening our previous findings.

The role of serotonin and its receptor in the pathogenesis of COPD has been investigated and is known to play an important role as an inflammatory mediator in pulmonary function [11,42,43]. In a COPD rodent model, simvastatin treatment reduced serotonin levels, suggesting that serotonin has an impact on the COPD-related inflammation [44]. This is the first study to report gender stratification in investigating serum serotonin levels, and the results further highlight the importance of including gender effects in the data analysis. It has been shown that the blood level of serotonin was negatively associated with age for both genders and positively associated with osteocalcin (non-collagenous protein associated with bone assembly), but only in young women; however, no hormonal association was observed [45]. An accumulation of T-cells has also been reported in the lungs of COPD patients, with a predominance of CD8+ T-cells in the small airways compared to the healthy population irrespective of smoking status [46]. We observed a positive association of serotonin with CD4+ T-cells in BAL and a negative association with CD8+ T-cells in female COPD smokers. There is a paucity of information on sex-differences in immune cells. It has been reported that females have higher levels of circulating CD4+ and CD8+ T-cells [47]. Negative associations also remained at the CD8+ T-cell subset level in females with COPD, suggesting a persistent inflammation in female COPD smokers. Anxiety and depressive symptoms are common in patients affected by COPD, even when their disease is mild in terms of forced expiratory volume in one second (FEV_1_) and respiratory symptoms in women [48]. We also observed a positive association between serotonin, a marker of stress, and female COPD smokers’ emotional function. In addition to serotonin, both kynurenine and kynurenic acid can exhibit immunomodulatory functions, which could be of interest in the immunopathology of COPD [49,50]. It would be of significant interest to quantify the tryptophan pathway in BAL fluid, which could potentially provide stronger associations with the BAL cytokine measurements.

This is the first study in which the effects of both smoking and gender on the tryptophan pathway were studied in relation to COPD. The result shows that (1) metabolite concentrations were primarily affected by smoking; (2) COPD-related effects were subtle, and (3) smoking cessation may be beneficial for normalizing serum IDO and TPH activity. One of the limitations of the current study is the small sample size of the COPD ex-smokers group; accordingly, these results should be regarded as preliminary. In addition, the overall relatively small size of the study suggests that findings should be interpreted with caution due to limited statistical power and certainly require validation in an independent cohort. However, the observed gender-differential in metabolite levels has been consistently observed in other studies performed with the Karolinska COSMIC cohort [23,24,25] as well as other COPD studies [41,51] and was recently reviewed [52]. It is accordingly advised that future works on COPD and potentially other obstructive lung diseases perform gender stratification when analyzing the findings. It would be of significant interest to examine the potential role of microbial metabolism of tryptophan in the observed metabolic profiles [53]. Inferred serum IDO activity was downregulated and serotonin levels were upregulated in smoking and in the smoking COPD population, specifically in males. A strong association of IDO activity with chemokines and cytokines, and serum serotonin with CD4+ and CD8+ T-cells in BAL with female COPD, again support previous findings that along with smoking, gender-based associations are prominent in the pathogenesis of COPD. These collective findings demonstrate that tryptophan metabolites show a gender-specific profile in COPD, suggest that disruptions in serotonin levels in COPD can be associated with depressive symptoms, and further support the need to perform gender stratification in COPD studies.

## 4. Materials and Methods

### 4.1. Subjects and Study Design

The COSMIC (www.clinicaltrials.gov/ct2/show/NCT02627872) cohort is a three-group cross sectional study. COPD subjects were defined by GOLD stage I–II/A-B; forced expiratory volume in one second (FEV_1_) = 51–97% and FEV1/ forced vital capacity (FVC) <70. Peripheral blood samples were collected from 39 healthy never-smokers, 40 smokers with normal lung function (hereafter referred as “smokers”), and 37 individuals with COPD, giving a total cohort size of 116. The COPD group consisted of both current smokers (*n* = 27, here after referred as “COPD smokers”) and ex-smokers (*n* = 11, >2 years since smoking cessation, hereafter referred as “COPD ex-smokers”). Groups were matched for age, gender, and current smoking status and history where relevant (Table 3). Participants had no history of allergy or asthma, did not use inhaled or oral corticosteroids, and had no exacerbations for at least 3 months prior to study inclusion. In vitro screenings for the presence of specific Immunoglobulin-E antibodies (Phadiatop, Stockholm, Sweden) were negative. Reversibility was tested after inhalation of two doses of 0.25 mg terbutaline (Bricanyl; Turbuhaler®; AstraZeneca, London, UK). Medications (including oral contraceptives, estrogen replacement, and Non-Steroidal Anti-Inflammatory Drugs (NSAIDs) or other potential lipid mediator-modifying drugs) were recorded by means of a questionnaire. Lung function parameters were calculated as post-bronchodilator percent of predicted using the European Community of Coal and Steel (ECCS) normal values. COPD patients and smokers were matched in terms of smoking history (>10 pack years) and current smoking habits (>10 cigarettes/day the past 6 months). The study was approved by the Stockholm Regional Ethical Board (COSMIC cohort: No. 2006/959-31/1) and participants provided their informed written consent.

### 4.2. Tryptophan Pathway Metabolites Measurement by LC-MS/MS

Tryptophan, d5-tryptophan, kynurenine, kynurenic acid, serotonin, and d4-serotonin were purchased from Sigma-Aldrich (St. Louis, MO, USA); d4-kynurenine and d5-kynurenic acid were acquired from Buchem BV (Apeldoorm, The Netherlands). The MS-grade formic acid, methanol, and acetonitrile were purchased from Sigma-Aldrich (St. Louis, MO, USA). Tryptophan metabolites were extracted from 50 µL of serum using solid phase extraction (SPE, Oasis® MAX, Waters Corporation, Milford, CT, USA). The solid phase was equilibrated with methanol and 5% ammonium hydroxide in milliQ-water followed by adding internal standard solution in 5% ammonium hydroxide, serum samples, and standards into the SPE-plate. The solid phase was washed with 5% ammonium hydroxide and analytes were eluted with 60% acetonitrile, 38% methanol, and 2% formic acid. The organic phase was evaporated using nitrogen and samples reconstituted in 2.5 % formic acid in milliQ water before analysis.

Then, 7.5 µL of the reconstituted extract were injected in a Waters Acquity ultrahigh performance LC (UPLC) system equipped with a HSST3 2.1 × 100 mm, 1.8-µm particle column. The mobile phases consisted of 2.1% formic acid in MilliQ water (A phase) and 0.1% formic acid in 95% acetonitrile (B phase). The gradient started at 2% B for 2 min following gradient to 10% at 2.5–4 min, 15% B at 7 min, and 90% B at 7.1–11 min at a flow rate of 300 µL/min. For tryptophan measurements the samples were further diluted with 1 mL milliQ-water and reinjected with a gradient of 10% B at 0–2.5 min, 30% B at 4 min, and 90% B at 4.5–8 min. Retention times for kynurenine, tryptophan, kynurenic acid, and serotonin were 3.9, 2.8, 6.4, and 3.7 min, respectively.

Metabolite detection was performed using a Waters Xevo TQ-S triple quadrupole mass spectrometer operating in positive ionization MS/MS configuration. The mass spectrometer was tuned for all analytes and the mass spectral transitions were set at *m/z* 209 > 146 (kynurenine), 205 > 91 (tryptophan), 190 > 116 (kynurenic acid), 177 > 115 (serotonin), and for the internal standards, 213 > 150 (d4-kynurenine), 210 > 150 (d5-tryptophan), 194 > 120 (d5-kynurenic acid), and 181 > 118 (d4-serotonin). Standards of each analyte were used to establish a linear calibration curve and plotted using the ratio of analyte peak area over internal standard peak area after integration by Masslynx 4.1 software (Waters Corporation, Milford, CT, USA).

The developed method was evaluated for analytical performance. Quality control (QC) Low and QCHigh samples were prepared using endogenous and spiked EDTA plasma at 1910, 50,400, 64.9, and 400 nmol/L for kynurenine, tryptophan, kynurenic acid, and serotonin, respectively. The coefficients of variance (CV) for the analytes in the QCLow and QCHigh material were: serotonin 5.8–5.9%, kynurenine 9–20%, tryptophan 18–20%, and kynurenic acid 4–20%, with a spike bias of within 17%. The lower LOQ for each metabolite was set based upon the standard curve, except for kynurenic acid, which was set to 0.015 µM (except when samples were diluted 2-fold where the value was instead set to 0.03 µM). Potential matrix effects were addressed by the use of isotopically labeled internal standards for each of the 4 metabolites. The SPE extraction yields were evaluated to be: serotonin 84–88%, kynurenine 82–92%, tryptophan 93–93%, and kynurenic acid 95–106%, covering the calibration range (Table 4).

### 4.3. Cytokine and Chemokine Measurements

A series of blood and BAL cells cytokines and chemokines were measured in the COSMIC cohort as previously described [24].

### 4.4. Measurement of IDO and TPH Activity

The ratios of kynurenine/tryptophan and serotonin/tryptophan have been proposed for use as indicators of serum IDO and TPH activity, respectively [54].

### 4.5. Statistical Analysis

All statistical analysis was performed in GraphPad Prism software (version 5.02, GraphPad Software, Inc., La Jolla, San Diego, CA, USA). Unless stated otherwise, categorical variables are expressed as numbers and continuous variables as medians (IQR). The data distribution was tested using a Shapiro–Wilk normality test. A Kruskal–Wallis ANOVA (*p* <0.05) was applied to compare the groups, and a non-parametric Mann–Whitney U test (*p* <0.05) was used to determine the significance between the groups. In all figures in both the main manuscript as well as supplemental information, *p*-values are only provided for those comparisons that reach the *p* <0.05 level of significance. Correlations were calculated using the Spearman’s rank correlation coefficient.

## Figures and Tables

**Table 1:** Serum concentration of the tryptophan pathway in the ‘COPD & Smoking from an “OMIC” Perspective’ (COSMIC) cohort including both genders.

Metabolite (µM)	Never-Smokers	Smokers	COPD Smokers	COPD Ex-Smokers	*p*-value
Tryptophan	67.2 (65.8, 77.6)	71.5 (61.6, 79.4)	63.1 (56.5, 68.9)	67.6 (54.6, 77.7)	0.2
Kynurenine	1.7 (1.5, 1.9)	1.5 (1.1, 1.6)	1.4 (1.3, 1.8)	1.9 (1.5, 2.0)	0.003
Kynurenic acid	0.04 (0.03, 0.04)	0.03 (0.02, 0.04)	0.04 (0.03, 0.04)	0.05 (0.04, 0.06)	0.009
Serotonin	0.5 (0.4, 0.75)	0.6 (0.5, 0.8)	0.7 (0.5, 0.9)	0.4 (0.3, 0.7)	0.03

The values are presented as median (interquartile range). The *p*-value was calculated using Kruskal–Wallis one-way ANOVA. COPD = chronic obstructive pulmonary disease.

**Table 2:** Correlation of estimated serum IDO activity with cytokines and chemokines from blood and bronchoalveolar lavage (BAL) cells.

Cytokines and Chemokines	COPD Joint Gender			COPD Females			COPD Males		
	XY Pairs	r	*p*-value	XY Pairs	*r*	*p*-value	XY Pairs	*r*	*p*-value
Blood cells									
CCL3	21	0.41	0.06	9	0.88	0.0016	12	0.01	1.0
CCL4	18	0.56	0.02	8	0.74	0.04	10	0.39	0.3
CXCL9	18	0.60	0.009	8	0.93	0.0009	10	0.26	0.5
CXCL10	18	0.43	0.07	8	0.88	0.004	10	0.08	0.8
CXCL12	17	0.43	0.09	9	0.73	0.02	8	−0.14	0.7
IL-1b	20	0.46	0.04	9	0.62	0.08	11	0.45	0.2
IL-12	19	0.40	0.09	9	0.67	0.05	10	−0.08	0.8
IL-13	19	0.46	0.04	8	0.81	0.01	11	-*	1.0
BAL cells									
CXCR4 on CD8	20	−0.37	0.1	8	−0.76	0.03	12	−0.03	0.9
FOXP3+ of CD4+	19	0.57	0.01	8	0.65	0.08	11	0.39	0.2

Statistical significance was determined using a non-parametric Mann-Whitney test. *No correlation value. CCL = chemokine (C-C motif) ligand, CXCL = CXC chemokine ligand, COPD = chronic obstructive pulmonary disease, IL = interleukin, CXCR = CXC chemokine ligand receptor, FOXP3 = forkhead box P3.

**Table 3:** Clinical parameters of individuals from the Karolinska COSMIC cohort included in the current study.

Parameters	Never-Smokers (*n* = 39)	Smokers (*n* = 40)	COPD Smokers (*n* = 27)	COPD Ex-Smokers (*n* = 10)
Gender distribution (number of males)	19	20	14	5
Age (years)	60.0 (51.5, 64.0)	52.5 (49.0, 57.8)^*^	59.5 (55.8, 63.0)^**^	63.5 (53.8, 65.0)^**^
BMI	25.6 (23.3, 28.9)	24.5 (22.1, 26.1)	24.1 (21.1, 27.0)	27.6 (22.7, 30.2)
Smoking (packyears)	N.A.	33.5 (28.3, 40.0)^*^	42.0 (35.8, 45.5)^*,**^	32.5 (21.5, 38.5)^*^
Current smoking/day	N.A.	17.0 (12.0, 20.0)	20.0 (14.0, 20.0)	N.A.
GOLD Stage (1/2)	N.A.	N.A.	12/14	5/5
GOLD-2011 (A/B/C)	N.A.	N.A.	19/7	6/3/1
Blood leukocytes (×10^9^/L)	5.6 (4.8, 6.8)	7.2 (6.6, 8.2)^*^	7.7 (6.2, 9.2)^*^	6.9 (6.5, 8.4)^*^
Blood platelets (×10^9^/L)	245.0 (216.0., 274.0)	252.5 (231.5, 298.5)	267.5 (237.3, 334.5)^*^	217.7 (197.3, 303.0)
Antitrypsin (g/L)	1.4 (1.3, 1.5)	1.5 (1.4, 1.7)	1.6 (1.4, 1.7)^*^	1.4 (1.3, 1.7)
FEV_1_(%)	116.0 (108.0, 121.0)	107.0 (95.5, 116.0)^*^	72.0 (63.5, 83.3)^*,**^	69.5 (63.8, 75.5)^*,**^
FEV_1_/FVC (%)	80.0 (76.0, 83.0)	76.0 (72.3, 78.8)^*^	60.0 (55.8, 65.0)^*,**^	59.0 (56.0, 62.8)^*,**^
Emphysema (No/Yes)	N.A.	22/18	6/20	2/8
CB (No/Yes)	N.A.	30/10	19/7	8/2

Definition of abbreviations: BMI = body mass index, CB = chronic bronchitis, COPD = chronic obstructive pulmonary disease, FEV_1_ = forced expiratory volume in one second, FVC = forced vital capacity, GOLD = Global Initiative for Obstructive Lung Disease, N.A. = not applicable. ^*^p <0.05 vs. healthy never-smokers. ^**^p <0.05 vs. healthy smokers. Values are presented as median and interquartile range (IQR).

**Table 4:** Linear range of the developed tryptophan metabolite method.

Analyte	Linear Range
Kynurenine	0.1–10.0 µM
Kynurenic acid	0.01–0.5 µM
Serotonin	0.0002–3.6 µM
Tryptophan	5–5000 µM

## Supplementary Materials

The following are available online at https://www.mdpi.com/2218-1989/9/10/212/s1; Figure S1: Serum tryptophan pathway metabolites identified from non-targeted platform in the Karolinska COSMIC cohort; Figure S2: Correlation of tryptophan and kynurenine signals from the original metabolomics study and the concentrations quantified with the newly developed LC-MS/MS method; Figure S3: Joint gender comparison of serum tryptophan pathway metabolites quantified by LC-MS/MS in the Karolinska COSMIC cohort; Figure S4. Estimated IDO activity according to COPD GOLD stage; Figure S5: Estimated serum TPH activity stratified by gender; Figure S6: Correlation of serum serotonin levels with T-cell populations in blood; Figure S7: Correlation of serum serotonin levels with T-cell populations from blood in COPD smokers.; Figure S8: Correlation of serum serotonin levels with emotional function in COPD smokers; Figure S9: Serum concentration of tryptophan in the four study groups stratified by gender.

## References

[1] Agusti A., Barnes P.J. (2012). Update in chronic obstructive pulmonary disease 2011. Am. J. Respir. Crit. Care Med..

[2] Hogg J.C., Paré P.D., Hackett T.-L. (2017). The Contribution of Small Airway Obstruction to the Pathogenesis of Chronic Obstructive Pulmonary Disease. Physiol. Rev..

[3] Rabe K.F., Watz H. (2017). Chronic Obstructive Pulmonary Disease. Lancet.

[4] Forsslund H., Mikko M., Karimi R., Grunewald J., Wheelock Å.M., Wahlström J., Sköld C.M. (2014). Distribution of T-cell subsets in BAL fluid of patients with mild to moderate COPD depends on current smoking status and not airway obstruction. Chest.

[5] Vargas-Rojas M.I., Ramírez-Venegas A., Limón-Camacho L., Ochoa L., Hernández-Zenteno R., Sansores R.H. (2011). Increase of Th17 cells in peripheral blood of patients with chronic obstructive pulmonary disease. Respir. Med..

[6] Barnes P.J. (2016). Inflammatory mechanisms in patients with chronic obstructive pulmonary disease. J. Allergy Clin. Immunol..

[7] Sun N., Wei X., Wang J., Cheng Z., Sun W. (2016). Caveolin-1 Promotes the Imbalance of Th17/Treg in Patients with Chronic Obstructive Pulmonary Disease. Inflammation.

[8] Shaykhiev R., Sackrowitz R., Fukui T., Zuo W.L., Chao I.W., Strulovici-Barel Y., Downey R.J., Crystal R.G. (2013). Smoking-induced CXCL14 expression in the human airway epithelium links chronic obstructive pulmonary disease to lung cancer. Am. J. Respir. Cell Mol. Biol..

[9] Le Floc’h N., Otten W., Merlot E. (2011). Tryptophan metabolism, from nutrition to potential therapeutic applications. Amino Acids.

[10] Agus A., Planchais J., Sokol H. (2018). Gut Microbiota Regulation of Tryptophan Metabolism in Health and Disease. Cell Host Microbe.

[11] Lau W.K.W., Chan-Yeung M.M.W., Yip B.H.K., Cheung A.H.K., Ip M.S.M., Mak J.C.W. (2012). The role of circulating serotonin in the development of chronic obstructive pulmonary disease. PLoS ONE.

[12] Yeung A.W.S., Terentis A.C., King N.J.C., Thomas S.R. (2015). Role of indoleamine 2,3-dioxygenase in health and disease. Clin. Sci..

[13] Meier M.A., Ottiger M., Vögeli A., Steuer C., Bernasconi L., Thomann R., Christ-Crain M., Henzen C., Hoess C., Zimmerli W. (2017). Activation of the Serotonin Pathway is Associated with Poor Outcome in COPD Exacerbation: Results of a Long-Term Cohort Study. Lung.

[14] Cervenka I., Agudelo L.Z., Ruas J.L. (2017). Kynurenines: Tryptophan’s metabolites in exercise, inflammation, and mental health. Science.

[15] Gál E.M., Sherman A.D. (1980). l-Kynurenine Its synthesis and possible regulatory function in brain. Neurochem. Res..

[16] Platten M., Nollen E.A.A., Röhrig U.F., Fallarino F., Opitz C.A. (2019). Tryptophan metabolism as a common therapeutic target in cancer, neurodegeneration and beyond. Nat. Rev. Drug Discov..

[17] Herraiz T., Chaparro C. (2005). Human monoamine oxidase is inhibited by tobacco smoke: β-carboline alkaloids act as potent and reversible inhibitors. Biochem. Biophys. Res. Commun..

[18] Hayaishi O. (1996). Utilization of Superoxide Anion by Indoleamine Oxygenase-Catalyzed Tryptophan and Indoleamine Oxidation. Adv. Exp. Med. Biol..

[19] Sage L.K., Fox J.M., Mellor A.L., Tompkins S.M., Tripp R.A. (2014). Indoleamine 2,3-Dioxygenase (IDO) Activity During the Primary Immune Response to Influenza Infection Modifies the Memory T Cell Response to Influenza Challenge. Viral Immunol..

[20] Maneechotesuwan K., Wongkajornsilp A., Adcock I.M., Barnes P.J. (2015). Simvastatin suppresses airway IL-17 and upregulates IL-10 in patients with stable COPD. Chest.

[21] Morecroft I. (2005). Functional Interactions between 5-Hydroxytryptamine Receptors and the Serotonin Transporter in Pulmonary Arteries. J. Pharmacol. Exp. Ther..

[22] Khalil A.A., Davies B., Castagnoli N. (2006). Isolation and characterization of a monoamine oxidase B selective inhibitor from tobacco smoke. Bioorg. Med. Chem..

[23] Naz S., Kolmert J., Yang M., Reinke S.N., Kamleh M.A., Snowden S., Heyder T., Levänen B., Erle D.J., Sköld C.M. (2017). Metabolomics analysis identifies sex-associated metabotypes of oxidative stress and the autotaxin-lysoPA axis in COPD. Eur. Respir. J..

[24] Forsslund H., Yang M., Mikko M., Karimi R., Nyrén S., Engvall B., Grunewald J., Merikallio H., Kaarteenaho R., Wahlström J. (2017). Gender differences in the t-cell profiles of the airways in COPD patients associated with clinical phenotypes. Int. J. COPD.

[25] Balgoma D., Yang M., Sjödin M., Snowden S., Karimi R., Levänen B., Merikallio H., Kaarteenaho R., Palmberg L., Larsson K. (2016). Linoleic acid-derived lipid mediators increase in a female-dominated subphenotype of COPD. Eur. Respir. J..

[26] Ubhi B.K., Riley J.H., Shaw P.A., Lomas D.A., Tal-Singers R., MacNeef W., Griffin J.L., Connor S.C. (2012). Metabolic profiling detects biomarkers of protein degradation in COPD patients. Eur. Respir. J..

[27] Maneechotesuwan K., Kasetsinsombat K., Wongkajornsilp A., Barnes P.J. (2013). Decreased indoleamine 2,3-dioxygenase activity and IL-10/IL-17A ratio in patients with COPD. Thorax.

[28] Lewis G.D., Ngo D., Hemnes A.R., Farrell L., Domos C., Pappagianopoulos P.P., Dhakal B.P., Souza A., Shi X., Pugh M.E. (2016). Metabolic Profiling of Right Ventricular-Pulmonary Vascular Function Reveals Circulating Biomarkers of Pulmonary Hypertension. J. Am. Coll. Cardiol..

[29] Hervé C., Beyne P., Jamault H., Delacoux E. (1996). Determination of tryptophan and its kynurenine pathway metabolites in human serum by high-performance liquid chromatography with simultaneous ultraviolet and fluorimetric detection. J. Chromatogr. B Biomed. Appl..

[30] Meier M.A., Ottiger M., Vögeli A., Steuer C., Bernasconi L., Thomann R., Christ-Crain M., Henzen C., Hoess C., Zimmerli W. (2017). Activation of the tryptophan/serotonin pathway is associated with severity and predicts outcomes in pneumonia: Results of a long-term cohort study. Clin. Chem. Lab. Med..

[31] Suzuki Y., Suda T., Yokomura K., Suzuki M., Fujie M., Furuhashi K., Hahimoto D., Enomto N., Fujisawa T., Nakamura Y. (2011). Serum activity of indoleamine 2,3-dioxygenase predicts prognosis of community-acquired pneumonia. J. Infect..

[32] Wendt C., Gulcev M., Reilly C., Griffin T., Broeckling C., Sandri B., Witthuhn B., Hodgson S., Woodruff P. (2016). Tryptophan catabolism in acute exacerbations of chronic obstructive pulmonary disease. Int. J. Chron. Obstruct. Pulm. Dis..

[33] Pertovaara M., Heliövaara M., Raitala A., Oja S.S., Knekt P., Hurme M. (2006). The activity of the immunoregulatory enzyme indoleamine 2,3-dioxygenase is decreased in smokers. Clin. Exp. Immunol..

[34] Hogg J.C. (2004). Pathophysiology of airflow limitation in chronic obstructive pulmonary disease. Lancet.

[35] Saetta M., Mariani M., Panina-Bordignon P., Turato G., Buonsanti C., Baraldo S., Bellettato C.M., Papi A., Corbetta L., Zuin R. (2002). Increased expression of the chemokine receptor CXCR3 and its ligand CXCL10 in peripheral airways of smokers with chronic obstructive pulmonary disease. Am. J. Respir. Crit. Care Med..

[36] Costa C., Rufino R., Traves S.L., Lapa E., Silva J.R., Barnes P.J., Donnelly L.E. (2008). CXCR3 and CCR5 chemokines in induced sputum from patients with COPD. Chest.

[37] Taylor M.W., Feng G.S. (1991). Relationship between interferon-gamma, indoleamine 2,3-dioxygenase, and tryptophan catabolism. FASEB J..

[38] Han M.L.K., Postma D., Mannino D.M., Giardino N.D., Buist S., Curtis J.L., Martinez F.J. (2007). Gender and chronic obstructive pulmonary disease: Why it matters. Am. J. Respir. Crit. Care Med..

[39] Baggiolini M., Dewald B., Moser B. (1993). lnterleukin-8 and Related Chemotactic Cytokines—CXC and CC Chemokines. Adv. Immunol..

[40] Rollins B.J., Walz A., Baggiolini M. (1991). Recombinant human MCP-1/JE induces chemotaxis, calcium flux, and the respiratory burst in human monocytes. Blood.

[41] Tam A., Churg A., Wright J.L., Zhou S., Kirby M., Coxson H.O., Lam S., Man S.F.P., Sin D.D. (2016). Sex differences in airway remodeling in a mouse model of chronic obstructive pulmonary disease. Am. J. Respir. Crit. Care Med..

[42] Olson T.P., Snyder E.M., Frantz R.P., Turner S.T., Johnson B.D. (2007). Repeat length polymorphism of the serotonin transporter gene influences pulmonary artery pressure in heart failure. Am. Heart J..

[43] Eddahibi S., Chaouat A., Morrell N., Fadel E., Fuhrman C., Bugnet A.S., Dartevelle P., Housset B., Hamon M., Weitzenblum E. (2003). Polymorphism of the Serotonin Transporter Gene and Pulmonary Hypertension in Chronic Obstructive Pulmonary Disease. Circulation.

[44] Sun W., Jiang X., Yuan L., Li P., Wang J., Wang P., Zhang L., Sun B. (2015). Effect of Simvastatin on 5-HT and 5-HTT in a Rat Model of Pulmonary Artery Hypertension. Cell. Physiol. Biochem..

[45] Wang Q., Chen D., Nicholson P., Cheng S., Alen M., Mao L., Cheng S. (2014). The Associations of Serum Serotonin with Bone Traits Are Age- and Gender-Specific. PLoS ONE.

[46] Saetta M., Baraldo S., Corbino L., Turato G., Braccioni F., Rea F., Cavallesco G., Tropeano G., Mapp C.E., Maestrelli P. (1999). CD8+ve cells in the lungs of smokers with chronic obstructive pulmonary disease. Am. J. Respir. Crit. Care Med..

[47] Xia H.J., Zhang G.H., Wang R.R., Zheng Y.T. (2009). The influence of age and sex on the cell counts of peripheral blood leukocyte subpopulations in Chinese rhesus macaques. Cell. Mol. Immunol..

[48] Di Marco F., Verga M., Reggente M., Maria Casanova F., Santus P., Blasi F., Allegra L., Centanni S. (2006). Anxiety and depression in COPD patients: The roles of gender and disease severity. Respir. Med..

[49] Wirthgen E., Hoeflich A., Rebl A., Günther J. (2018). Kynurenic Acid: The Janus-faced role of an immunomodulatory tryptophan metabolite and its link to pathological conditions. Front. Immunol..

[50] Van der Leek A.P., Yanishevsky Y., Kozyrskyj A.L. (2017). The kynurenine pathway as a novel link between allergy and the gut microbiome. Front. Immunol..

[51] Tam A., Bates J.H.T., Churg A., Wright J.L., Man S.F.P., Sin D.D. (2016). Sex-related differences in pulmonary function following 6 months of cigarette exposure: Implications for sexual dimorphism in mild COPD. PLoS ONE.

[52] Han M.L.K., Arteaga-Solis E., Blenis J., Bourjeily G., Clegg D.J., DeMeo D., Duffy J., Gaston B., Heller N.M., Hemnes A. (2018). Female sex and gender in lung/sleep health and disease: Increased understanding of basic biological, pathophysiological, and behavioral mechanisms leading to better health for female patients with lung disease. Am. J. Respir. Crit. Care Med..

[53] Roager H.M., Licht T.R. (2018). Microbial tryptophan catabolites in health and disease. Nat. Commun..

[54] Widner B., Werner E.R., Schennach H., Wachter H., Fuchs D. (1997). Simultaneous measurement of serum tryptophan and kynurenine by HPLC. Clin. Chem..

