# Supplementary material for: Dysregulation of the Tryptophan Pathway Evidences Gender Differences in COPD
Source: Metabolites. 2019 Oct 1;9(10):212. doi: 10.3390/metabo9100212 (PMC6835831; doi:10.3390/metabo9100212)
Supplement: Supplementary file 1 [file metabolites-09-00212-s001.pdf]

metabolites

Supplementary Material

Dysregulation of the tryptophan pathway evidences gender differences in COPD

Shama Naz[1], Maria Bhat[2,3], Sara Ståhl[2,3], Helena Forsslund[4], C. Magnus Sköld[4], Åsa M. Wheelock[4,*], Craig E. Wheelock[1,*]

[1] Division of Physiological Chemistry 2, Department of Medical Biochemistry and Biophysics, Karolinska Institutet, Stockholm, Sweden
[2] AstraZeneca, Research and Development, Innovative Medicines, Personalised Healthcare and Biomarkers, Translational Science Centre, Science for Life Laboratory, Solna, Sweden
[3] Department of Clinical Neuroscience, Karolinska Institutet, Stockholm, Sweden
[4] Respiratory Medicine Unit, Department of Medicine Solna & Center for Molecular Medicine, Karolinska Institutet, Stockholm, Sweden

* Correspondence:
Craig E. Wheelock, PhD, e-mail@ craig.wheelock@ki.se; Tel.: +46 8 524 87630
Åsa Wheelock, PhD, e-mail@ asa.wheelock@ki.se; Tel.: +46 70 2200308

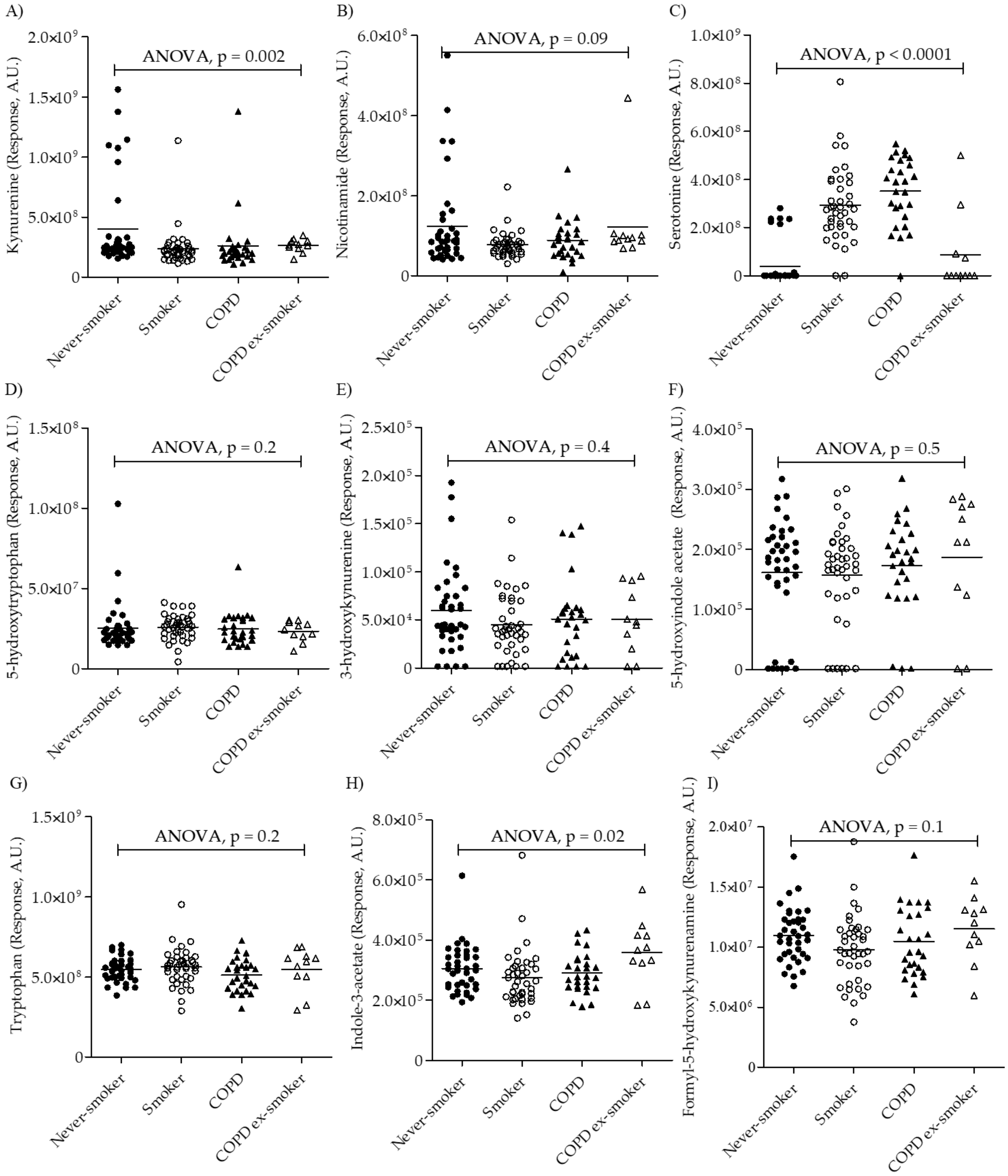

Figure S1. Serum tryptophan pathway metabolites identified from the non-targeted metabolomics study in the Karolinska COSMIC cohort. Comparison of (A) kynurenine, (B) nicotinamide, (C) serotonin, (D) 5-hydroxytryptophan, (E) 3-hydroxykynurenine, (F) 5-hydroxyindoleacetate, (G) tryptophan, (H) indole-3-acetate and (I) folmyl-5-hydroxykynurenamine abundances in healthy (Never-smokers, filled circles), smokers (open circles), COPD individuals (filled triangles) and COPD ex-smokers (open triangles). Significance was tested by applying a non-parametric Kruskal-Wallis one-way ANOVA.

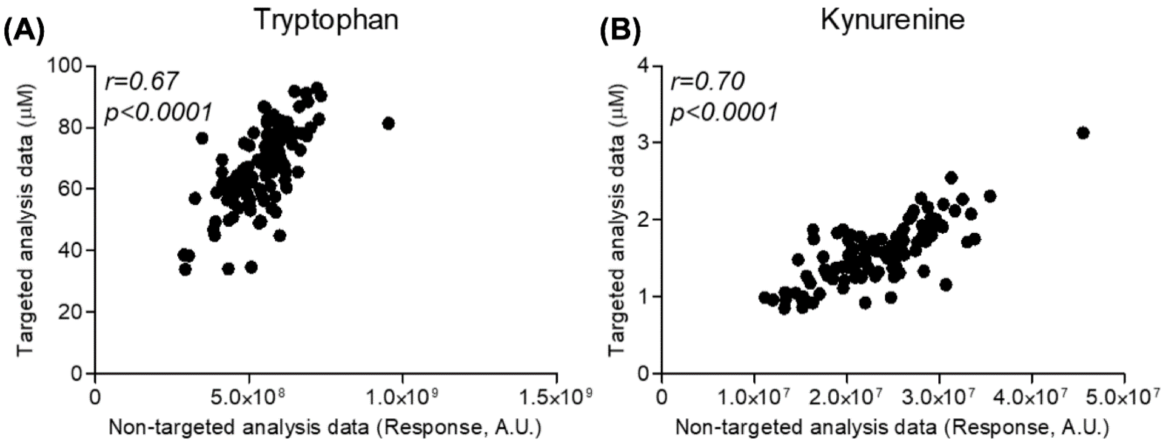

Figure S2. Correlation of (A) tryptophan and (B) kynurenine signals from the non-targeted metabolomics platform with the quantified concentrations acquired from the targeted LC-MS/MS method.

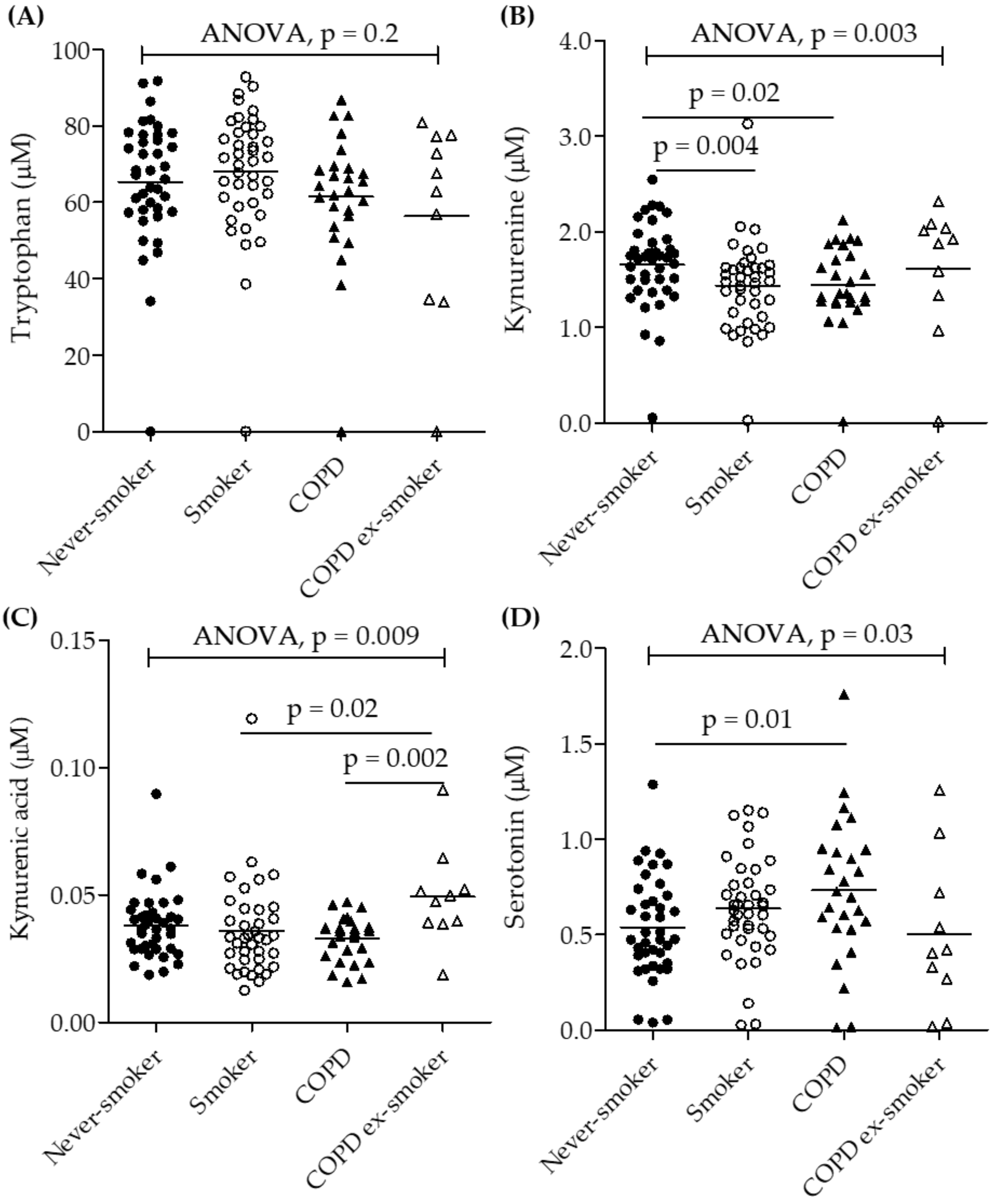

Figure S3. Joint gender comparison of serum tryptophan pathway metabolites quantified by LC-MS/MS in the Karolinska COSMIC cohort. Comparison of (A) tryptophan, (B) kynurenine, (C) kynurenic acid and (D) serotonin, in healthy (Never-smokers, filled circles), smokers (open circles), COPD individuals (filled triangles) and COPD ex-smokers (open triangles). Significance was tested by applying non-parametric Kruskal-Wallis one-way ANOVA and Mann-Whitney test.

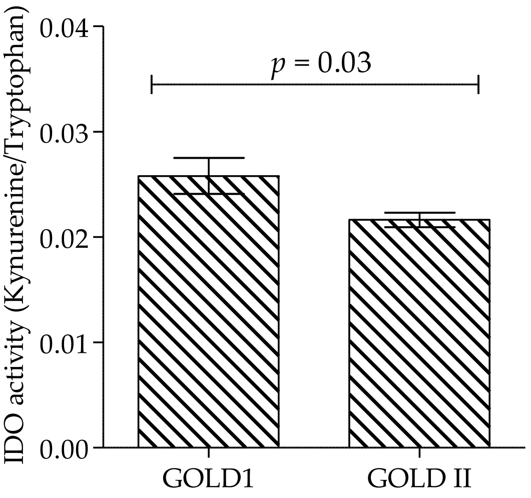

Figure S4. Estimated IDO activity according to COPD GOLD stage. Significance was tested by applying a non-parametric Mann-Whitney test.

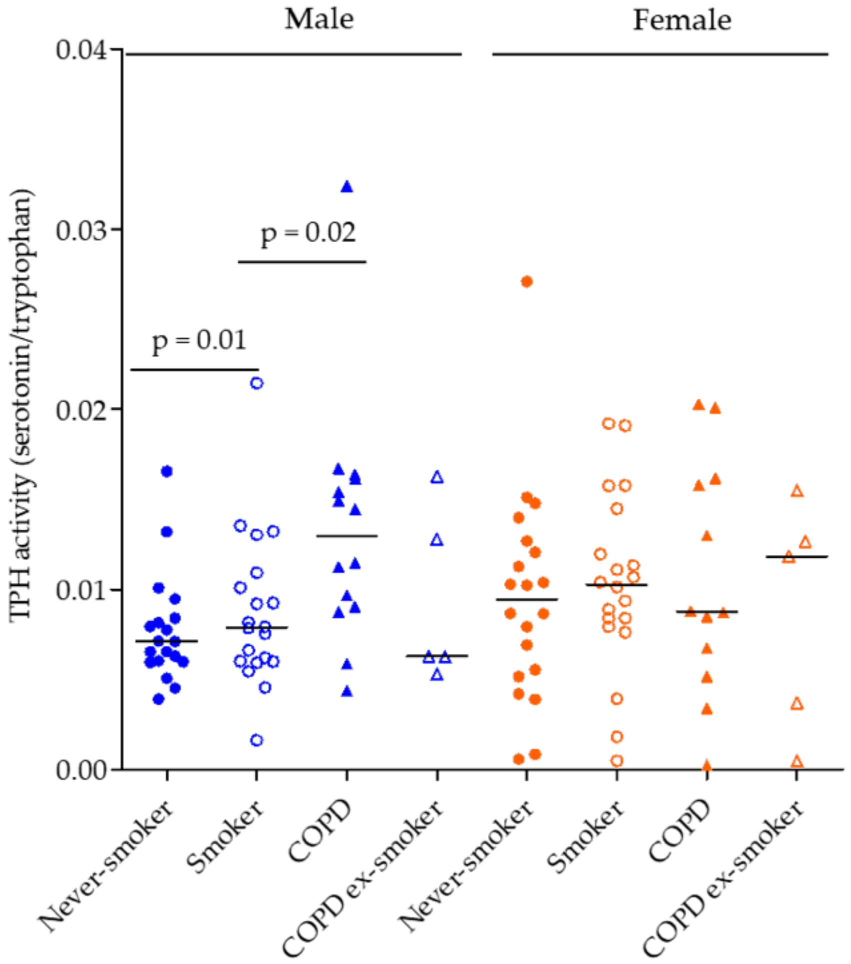

Figure S5. Estimated serum TPH (serotonin/tryptophan) activity, stratified by gender. Never-smokers: closed circles, Smokers: open circles, COPD smokers: closed triangles, COPD ex-smokers: open triangles, male: blue, female: orange. Significance was tested by applying a non-parametric Mann-Whitney test.

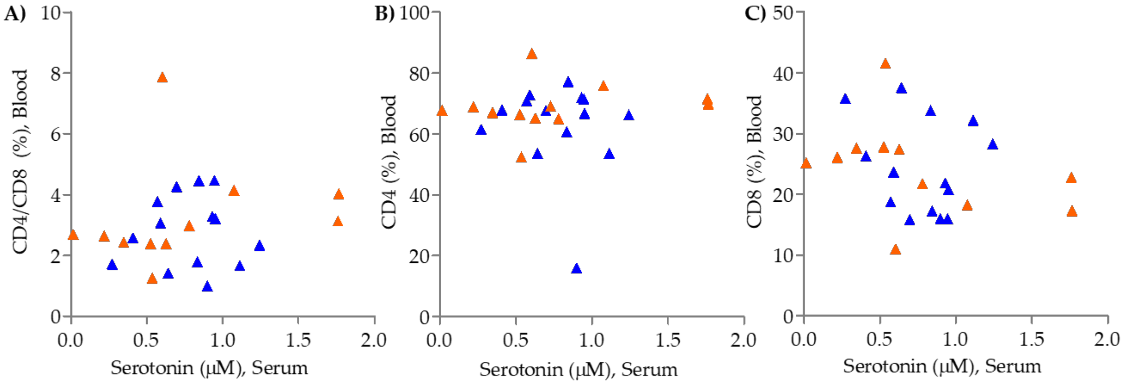

Figure S6: Correlation of serum serotonin levels with T cells populations from blood in COPD smokers. A) CD4/CD8 %, B) CD4 %, C) CD8 %. Male: blue triangles, female: orange triangles.

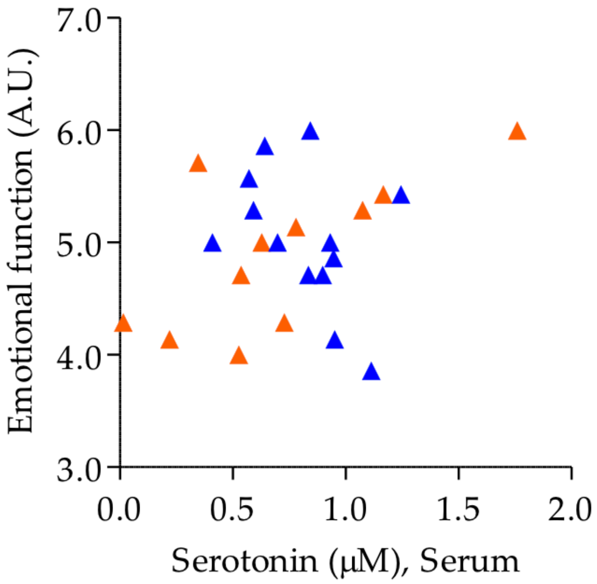

Figure S7. Correlation of serum serotonin levels with emotional function in COPD smokers. Male: blue triangles, female: orange triangles. Serotonin levels showed a positive association with emotional function in female COPD smokers (p= 0.03, r=0.64), but not males (p=0.1, r=-0.45).

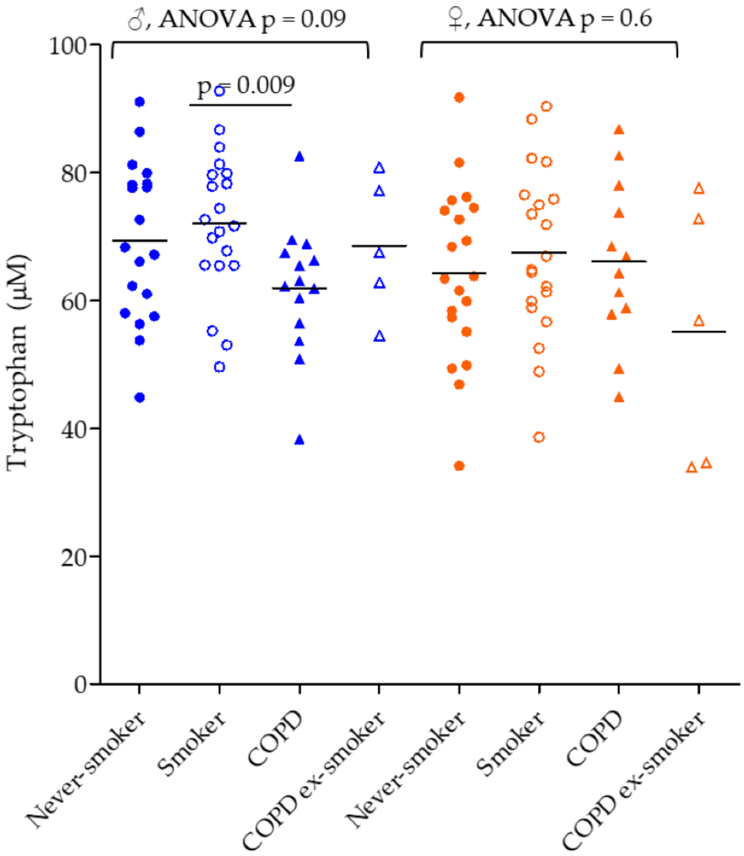

Figure S8. Serum concentration of tryptophan in four study groups, stratified by gender. Never-smokers: closed circles, Smokers: open circles, COPD smokers: closed triangles, COPD ex-smokers: open triangles, male: blue, female: orange. Significance was tested by applying a non-parametric Kruskal-Wallis ANOVA and a non-parametric Mann-Whitney test.
